# Coffee Consumption and Risk of Dementia and Alzheimer’s Disease: A Dose-Response Meta-Analysis of Prospective Studies

**DOI:** 10.3390/nu10101501

**Published:** 2018-10-14

**Authors:** Susanna C. Larsson, Nicola Orsini

**Affiliations:** 1 Unit of Nutritional Epidemiology, Institute of Environmental Medicine, Karolinska Institutet, 17177 Stockholm, Sweden; 2 Department of Public Health Sciences, Karolinska Institute, 17177 Stockholm, Sweden; nicola.orsini@ki.se

**Keywords:** Alzheimer’s disease, coffee, dementia, meta-analysis, prospective studies

## Abstract

Coffee consumption is associated with a reduced risk of several diseases but uncertainty remains about the influence of coffee consumption on the risk of dementia. We performed a dose-response meta-analysis to summarize the prospective data on coffee consumption and associated risk of dementia and Alzheimer’s disease. We identified studies by searching PubMed (from January 1966) and Web of Science (from January 1945) through 4 October 2018 and by scrutinizing the reference lists of pertinent publications. Two researchers independently reviewed the literature. Results were combined using a restricted cubic spline random-effects dose-response meta-analysis based on a one-stage approach. Eight relevant prospective studies were identified. These studies included 7486 dementia cases diagnosed among 328,885 individuals during an average follow-up of 4.9–25 years. Meta-analysis of all eight studies indicated no statistically significant association between coffee consumption and the risk of dementia and no deviations from a linear trend (*p* = 0.08). The relative risk of dementia per 1 cup/day increment of coffee consumption was 1.01 (95% confidence interval (CI) 0.98–1.05; *p* = 0.37). Meta-analysis of five studies that focused on Alzheimer’s disease revealed no association between coffee consumption and Alzheimer’s disease and no deviations from a linear trend (*p* = 0.79). The relative risk of Alzheimer’s disease per 1 cup/day increment of coffee consumption was 1.01 (95% confidence interval 0.95–1.07; *p* = 0.80). These results do not support an association between coffee consumption and an increased risk of overall dementia or Alzheimer’s disease specifically, but further research on the association of coffee consumption with dementia risk is needed.

## 1. Introduction

Dementia is an increasing global health concern due to the aging population. Globally, about 50 million individuals had dementia in 2015 and this number is projected to increase to 132 million by 2050 [1]. The predominant cause of dementia is Alzheimer’s disease (AD), which accounts for an estimated 60% or more of all cases [2]. The pathological hallmarks of AD are the deposition of amyloid-β (plaques) outside neurons and twisted strands of the protein tau inside neurons [2]. The identification of modifiable factors that can reduce the incidence of dementia is of high priority. 

Evidence from observational studies indicates that coffee consumption may lower the risk of several diseases, including diabetes, stroke, coronary heart disease, certain cancers, Parkinson’s disease and gout [3,4,5,6,7]. Coffee is the primary source of caffeine in most populations and contains phenolics and other bioactive compounds with potential beneficial or adverse effects on health. Experimental evidence indicates that caffeinated coffee and caffeine, which readily crosses the blood brain barrier [8], may influence the processes associated with AD. For example, experimental studies in rodents have demonstrated that caffeine and caffeinated coffee suppresses brain amyloid-β production [9,10,11], causes microglia activation [12], reduces hippocampal pro-inflammatory cytokines [9], protects against any dysfunction in the blood–brain barrier [13] and prevents memory impairment [11]. Thus, coffee consumption might influence the risk of AD and dementia but both observational studies testing this hypothesis and meta-analyses summarizing the data have yielded inconclusive results [14,15]. One meta-analysis reported an inverse association between coffee consumption (highest category or any consumption vs. lowest category or no coffee consumption) and the risk of AD but not dementia [14]. Another meta-analysis found that low (1–2 cups/day vs. <1 cup/day) but not high (>3 cups/day) coffee consumption was associated with a significantly reduced risk of AD and dementia [15]. In contrast, a Mendelian randomization found suggestive evidence of a positive association of a genetic predisposition to consume more coffee with AD risk [16]. 

To further clarify whether coffee drinking is related to the risk of dementia or AD, we performed a dose-response meta-analysis of prospective studies. This meta-analysis includes data from three additional prospective studies [17,18,19], which were not included in prior meta-analyses [14,15].

## 2. Methods

### 2.1. Search Strategy 

**Figure 1:**
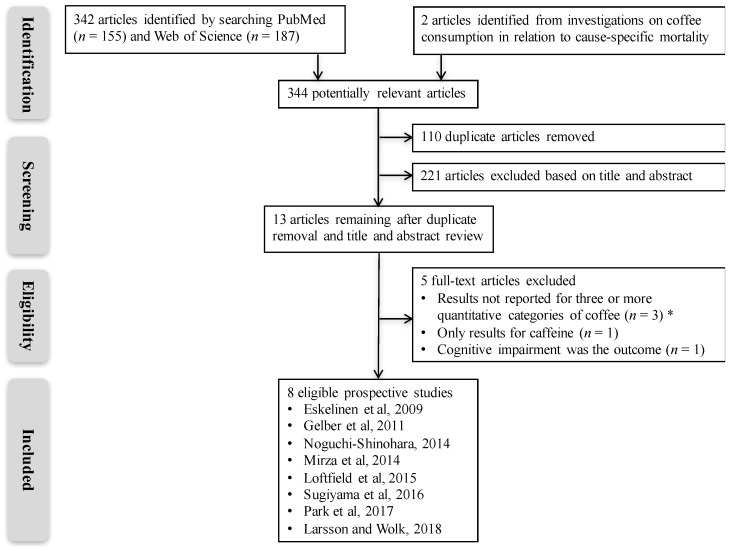
Summary of literature search. * Results reported for regular versus no regular coffee intake [20]; daily versus no daily coffee intake [21]; or per times/week increase in coffee intake [22].

The PRISMA guidelines were followed for this meta-analysis. We searched PubMed from 1 January 1966 through 4 October 2018, using the search terms “coffee” (All Fields) and “dementia” (All Fields) or “coffee” (All Fields) and “Alzheimer’s disease” (All Fields). Moreover, Web of Science was searched from 1 January 1945 through 4 October 2018 using the search query “TOPIC: (coffee) and TOPIC: (dementia) or TOPIC: (coffee) and TOPIC: (Alzheimer’s disease)”. We searched for additional studies by scrutinizing the reference lists of relevant articles. Two researchers (S.C.L. and N.O.) independently reviewed the literature. A summary of the literature search, screening and study selection is presented in Figure 1. 

### 2.2. Inclusion and Exclusion Criteria 

The eligibility criteria in this meta-analysis included prospective studies that reported relative risk (RR) estimates with corresponding 95% confidence intervals (CI), or sufficient data to estimate these, of AD or all-cause dementia for at least three categories of coffee consumption. Cross-sectional, case-control and Mendelian randomization studies as well as studies reporting results on cognitive impairment only were excluded. 

### 2.3. Data Extraction 

From each study, the authors (S.C.L. and N.O.) extracted the following information: study name and country in which the study was performed, sample size, number of cases, how cases were ascertained, age of participants, proportion of men, duration of follow-up, potential risk factors adjusted for in the multivariable analysis, the RRs with their 95% CI for each exposure category and the number of cases and total number of participants or person-years in each category. We extracted the multivariable RRs that were adjusted for the largest number of possible confounders. 

### 2.4. Assessment of Study Quality

The Newcastle-Ottawa Scale was used to assess study quality [23]. The assessment was based on selection, comparability and outcome. Details of the criteria are available in Figure S1. Studies received a score for each criteria met. The score ranged from 0 to 9, with a higher score indicating higher quality.

### 2.5. Statistical Analysis

Our primary analysis included dementia as the outcome (either all-cause dementia or AD if data on any dementia were not available in the study) according to the quantitative categories of coffee consumption. Studies that reported results on AD only were included in the primary analysis but were excluded in a sensitivity analysis. In addition, we performed analyses of coffee consumption in relation to AD specifically. We performed a random-effects dose-response meta-analysis using a one-stage approach and restricted maximum likelihood estimation [24]. We assessed potential departure from linearity using restricted cubic splines and applied a Wald-type test to detect departure from a simpler linear function [25]. The between-study heterogeneity was investigated with Cochran’s Q test and the *I*^2^ statistic [26]. Publication bias was assessed using Egger’s test and funnel plot [27]. The statistical analyses were conducted using the drmeta, metabias and metafunnel commands for Stata, version 14.2 (StataCorp, Texas, USA) 

## 3. Results

Eight prospective studies that examined the association of coffee consumption with the risk of AD and/or all-cause dementia were eligible for inclusion in the current meta-analysis [17,18,19,28,29,30,31,32] (Figure 1). The study characteristics are shown in Table 1. Three studies were conducted in the United States, three in Europe and two in Japan. All but one study included both men and women. Combined, the eight studies included 7486 cases of dementia (including AD cases specifically for two studies that did not provide results on all-cause dementia [17,18]) diagnosed among 328,885 individuals during an average follow-up of 4.9–25 years. Coffee consumption in the highest category ranged from every day in a Japanese study [30] to 5 or more cups/day in Finnish and Swedish cohorts [19,28]. Study quality, which was assessed based on the 9-stars Newcastle-Ottawa Scale, ranged from 7 to 9 (mean of 7.6) (Table 1).

**Figure 2:**
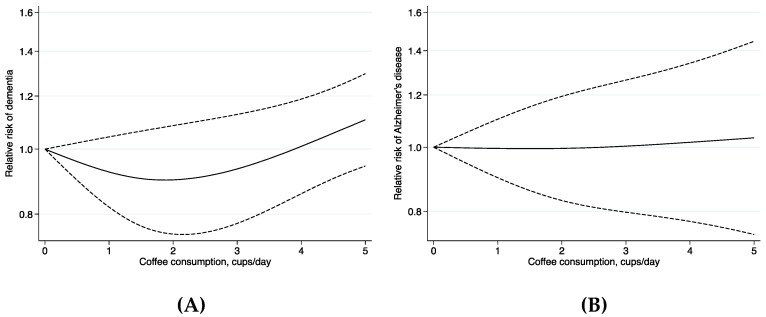
Dose-response association of coffee consumption with risk of dementia (**A**) and Alzheimer’s disease (**B**). The analyses of dementia and Alzheimer’s disease include eight and five prospective studies, respectively.

Meta-analysis combining the data from the eight studies revealed no statistically significant association between coffee consumption and the risk of dementia and no deviations from a linear trend (*p* = 0.08) (Figure 2). The RRs (95% CI) of dementia were 0.92 (0.82–1.04) for 1 cup/day, 0.90 (0.75–1.08) for 2 cups/day, 0.93 (0.77–1.13) for 3 cups/day, 1.01 (0.86–1.19) for 4 cups/day and 1.11 (0.94–1.30) for 5 cups/day of coffee consumption (Figure 2). The RR of dementia per 1 cup/day increment of coffee consumption was 1.01 (95% CI 0.98–1.05; *p* = 0.37). There was no heterogeneity between the results of individual studies (*I*^2^ = 30.7%, *p* = 0.18) and no indication of publication bias (*p* = 0.48) (funnel plot is presented in Figure S2). Exclusion of two studies with AD mortality as the outcome [17,18] did not change the results appreciably. The RR of dementia in a meta-analysis that combined the results of the remaining six studies was 1.01 (95% CI 0.98–1.03; *p* = 0.58) per 1 cup/day increment of coffee consumption.

Meta-analysis of the five studies with results on AD revealed no association between coffee consumption and the risk of AD and there was no indication of departure from linearity (*p* = 0.79) (Figure 2). The RR of AD per 1 cup/day increment in coffee consumption was 1.01 (95% CI 0.95–1.07; *p* = 0.80), with no statistically significant between-study heterogeneity (*I*^2^ = 41.8%, *p* = 0.14).

## 4. Discussion

This contemporary meta-analysis of eight prospective studies provides no evidence that coffee consumption is associated with the risk of AD or overall dementia. However, we cannot rule out that we may have overlooked a weak association.

Three studies reporting results on coffee consumption and AD risk were non-eligible for inclusion in this dose-response meta-analysis because results were not reported in different quantitative categories of coffee consumption [20,21,22]. Two of those studies were consistent in finding a lack of association between coffee consumption and the risk of AD [20,22]. In a cohort of 2622 older German adults, including 418 AD cases diagnosed during 10 years of follow-up, the hazard ratio of AD per one time/week increase in coffee consumption was 0.97 (95% CI 0.90–1.04) [22]. Likewise, in a cohort of 694 Canadian adults, including 36 AD cases diagnosed during 5 years of follow-up, the RR of AD for regular versus no regular coffee consumption was 1.03 (95% CI 0.47–2.30) [20]. Results from another cohort of 4615 Canadian adults, including 194 AD cases diagnosed over 5 years, showed that daily versus no daily coffee consumption was associated with a reduction of 31% in the risk of AD [21]. There is also no support from Mendelian randomization studies that a genetic predisposition to consume more coffee is associated with a decreased risk of AD (if anything, a positive association has been observed) [16] or that coffee/caffeine intake is associated with cognitive decline [33].

A strength of this meta-analysis based on the data from eight prospective studies is that the association between coffee consumption and the risk of dementia could be assessed with relatively high precision. Furthermore, we were able to investigate the dose-response relationship between coffee consumption and dementia risk. A limitation of this meta-analysis is that self-reported coffee consumption is subject to measurement errors, which may have resulted in attenuated results. The exact amount and concentration of coffee consumed are difficult to measure by a questionnaire and therefore, this meta-analysis may not have captured the true dose-response relationship between coffee consumption and dementia risk. Likewise, the diagnosis of dementia and AD differed among studies and some degree of misclassification of dementia is likely to have occurred in most studies. Another shortcoming is that the findings were based on data from observational studies and thus, residual confounding and reverse causality cannot be ruled out as explanations for the lack of an observed association of coffee consumption with dementia risk. Finally, we were unable to examine whether the intake of caffeinated or decaffeinated coffee specifically is associated with the risk of dementia. 

## 5. Conclusions

In conclusion, the available data from observational prospective studies provide no evidence that coffee consumption is associated with the risk of AD or all-cause dementia. Nevertheless, there have only been a few studies examining the relationship of coffee consumption with the risk of dementia and AD specifically are relatively few, while the misclassification of coffee consumption and of dementia and may have attenuated the results. Therefore, additional studies that are well conducted are necessary to establish whether coffee consumption influences the risk of dementia or AD.

## Figures and Tables

**Table 1:** Prospective studies of coffee consumption and risk of Alzheimer’s disease and all-cause dementia.

Author, Year (reference)	Study Name, Country	Ascertainment of AD and Dementia	Cases (Total Sample Size)	Age; % Men	Follow-up Time	Adjustment for Confounders	NOS Score *	Coffee Intake Category	RR (95% CI) of AD	RR (95% CI) of Dementia
Eskelinen et al., 2009 [28]	Cardiovascular Risk Factors, Aging and Dementia study, Finland	Neurological and clinical examination	58 AD, 61 dementia (1409)	65–79 years; 62	21 years (mean)	Age, sex, education, area of residence, smoking, BMI, systolic blood pressure, total cholesterol, ApoE ε4 status	7	0–2 cups/day 3–5 cups/day >5 cups/day	Reference 0.42 (0.12–1.46) 1.01 (0.33–3.08)	Reference 0.30 (0.10–0.93) 0.83 (0.32–2.15)
Gelber et al., 2011 [29]	Honolulu-Asia Aging Study, United States (Hawaii)	Neurological and clinical examination	118 AD, 226 dementia (3494)	71–93 years; 100	25 years	Age, education, smoking, physical activity, elevated cholesterol, hypertension, ApoE ε4 status	9	0 oz/day 4–8 oz/day 12–16 oz/day 20–24 oz/day ≥28 oz/day	Reference 0.89 (0.50–1.59) 1.09 (0.60–2.00) 0.95 (0.45–2.00) 0.59 (0.23–1.54)	Reference 0.93 (0.59–1.46) 1.24 (0.78–1.97) 1.14 (0.66–1.98) 1.09 (0.59–2.00)
Noguchi-Shinohara et al., 2014 [30]	Nakajima project, Japan	Neurological and clinical examination	26 dementia (490)	>60 years; ~35	4.9 years (mean)	Age, sex, education, smoking, hypertension, diabetes, hyperlipidemia, ApoE ε4 status, physical activities and/or other hobbies, alcohol, tea	7	None 1–6 days/week Every day	NA	Reference 1.00 (0.34–2.99) 0.70 (0.22–2.17)
Mirza et al., 2014 [31]	Rotterdam Study, The Netherlands	Neurophysiological testing and linkage to medical records	814 dementia (5408)	≥55 years; 59	13.2 years (mean)	Age, sex, education, smoking, BMI, hypertension, diabetes, family history of dementia, working status, alcohol	9	0–1 cup/day >1–3 cups/day >3 cups/day	NA	Reference 0.88 (0.67–1.16) 1.00 (0.76–1.30)
Loftfield et al., 2015 [17]	Prostate, Lung, Colorectal and Ovarian Screening Trial, United States	Linkage to the National Death Index	93 AD deaths (90,317)	55–74 years; NA	12 years	Age, sex, education, marital status, race/ethnicity, smoking, BMI, diabetes, supplemental vitamin use, ibuprofen, aspirin, menopausal hormone therapy, and intake of alcohol, energy, red and processed meat, white meat, saturated fat, fruit, and vegetables	7	0 cup/day <1 cup/day 1 cup/day 2–3 cups/day ≥4 cups/day	Reference 1.01 (0.53–1.95) 0.66 (0.32–1.36) 0.59 (0.31–1.11) 0.72 (0.33–1.58)	NA
Sugiyama et al., 2016 [32]	Ohsaki Cohort, Japan	Linkage to the Long-term Care Insurance database	1107 dementia (13,137)	≥65 years; ~45	5.7 years	Age, sex, education, smoking, BMI, walking duration, history of stroke, hypertension, myocardial infarction, diabetes, arthritis, osteoporosis, and fracture, psychological distress, participating in any community activities, alcohol, green tea	7	Never Occasionally 1–2 cups/day ≥3 cups/day	NA	Reference 0.73 (0.62–0.86) 0.72 (0.61–0.84) 0.82 (0.65–1.02)
Park et al., 2017 [18]	Multiethnic Cohort, United States	Linkage to the death register	1404 AD deaths (185,855)	45–75 years; ~45	16.2 years (mean)	Age, sex, ethnicity, education, smoking, preexisting illness, BMI, physical activity, and intake of alcohol, total energy, and energy from fat	7	None 1–3 cups/month 1–6 cups/week 1 cup/day 2–3 cups/day ≥4 cups/day	Reference 1.01 (0.72–1.41) 0.92 (0.69–1.24) 0.90 (0.71–1.14) 1.16 (0.90–1.49) 1.33 (0.86–2.04)	NA
Larsson and Wolk, 2018 [19]	Swedish Mammography Cohort and Cohort of Swedish Men, Sweden	Linkage to national patient register	1299 AD, 3755 dementia (28,775)	65–83 years; 53	12.6 years (mean)	Age, sex, education, smoking, BMI, exercise, walking or bicycling, history of hypertension, hypercholesterolemia, or diabetes, sleep duration, alcohol, DASH diet	8	<1 cup/day 1–2.9 cups/day 3–4.9 cups/day ≥5 cups/day	Reference 0.90 (0.70–1.17) 1.01 (0.78–1.30) 0.93 (0.70–1.24)	Reference 0.99 (0.85–1.16) 1.03 (0.88–1.21) 1.07 (0.90–1.28)

AD, Alzheimer’s disease; BMI, body mass index; CI confidence interval; DASH, Dietary Approaches to Stop Hypertension; oz; ounce; RR, relative risk; NA, not available.

* Newcastle-Ottawa Scale (NOS) for assessment of study quality; the score ranges from 0–9, with higher score indicating higher quality.

## Supplementary Materials

The following are available online at http://www.mdpi.com/2072-6643/10/10/1501/s1, Figure S1: Newcastle-Ottawa Scale: Details of how the criteria were applied; Figure S2: Funnel plot to visually assess publication bias. Egger’s test: *p* = 0.48.
