# Supplementary material for: Coffee Consumption and Risk of Dementia and Alzheimer’s Disease: A Dose-Response Meta-Analysis of Prospective Studies
Source: Nutrients. 2018 Oct 14;10(10):1501. doi: 10.3390/nu10101501 (PMC6213481; doi:10.3390/nu10101501)
Supplement: Supplementary file 1 [file nutrients-10-01501-s001.zip › Supplement.pdf]

Figure S1. Newcastle-Ottawa Scale: Details of how the criteria were applied

Selection

1) Representativeness of the exposed cohort

- ❖ Star assigned if exposed cohort was truly or somewhat representative of the average coffee consumers in the community (i.e., the sample was random or covered all individuals residing in one or a few geographical areas).

2) Selection of the non-exposed cohort

- ❖ Star assigned where non-exposed participants were drawn from the same population as the exposed participants.

3) Ascertainment of exposure

- ❖ Star assigned if coffee consumption had been assessed using a structured interview.

4) Demonstration that outcome of interest was not present at the start of study

- ❖ Star assigned if participants with dementia before baseline were excluded.

Comparability

1) Comparability of cohorts on the basis of the design or analysis

- ❖ One star assigned to studies that adjusted for age and sex (if analysis included both men and women).
- ❖ Second star assigned if further adjustments were made for education and smoking.

Outcome

1) Assessment of outcome

- ❖ Star assigned where an assessment for dementia was made through a non-subjective method (i.e., neurological test or via linkage to national register). No star assigned to studies based on self-reported dementia.

2) Was follow-up long enough for outcomes to occur?

- ❖ Star assigned where follow-up was at least 10 years.

3) Adequacy of follow up of cohorts

- ❖ Any loss to follow-up was assumed to be random and not related to coffee consumption.

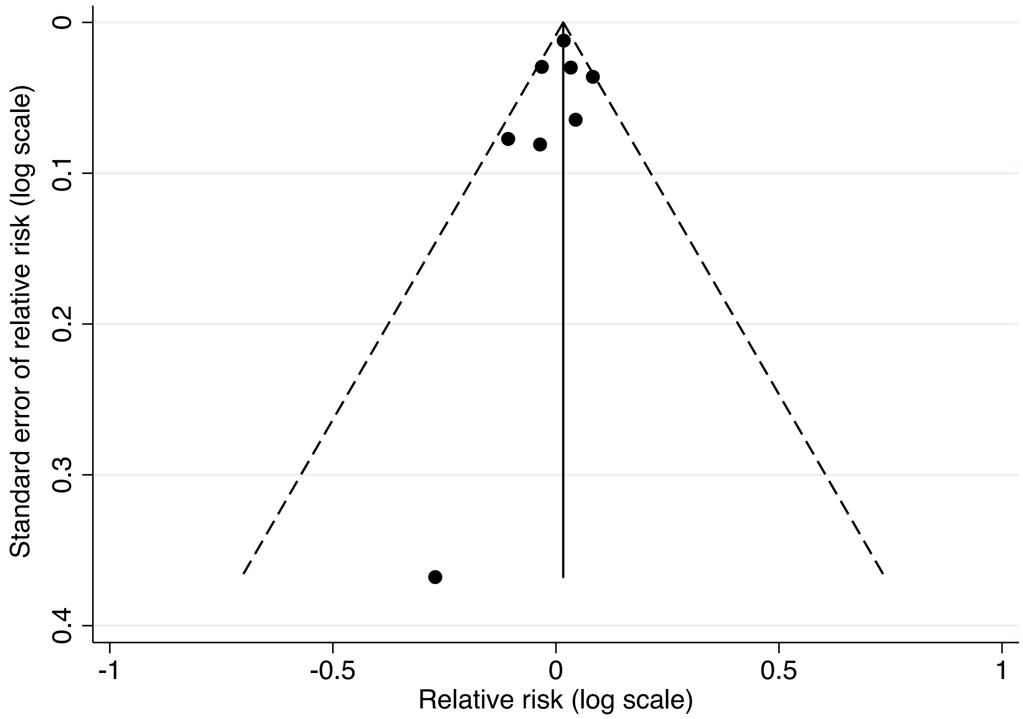

Figure S2. Funnel plot to visually assess publication bias. Egger's test: p = 0.48.
